# Sheep Wool δ^13^C Reveals No Effect of Grazing on the C_3_/C_4_ Ratio of Vegetation in the Inner Mongolia–Mongolia Border Region Grasslands

**DOI:** 10.1371/journal.pone.0045552

**Published:** 2012-09-27

**Authors:** Karl Auerswald, Max H.O.M. Wittmer, Radnaakhand Tungalag, Yongfei Bai, Hans Schnyder

**Affiliations:** 1 Lehrstuhl für Grünlandlehre, Technische Universität München, Alte Akademie 12, Freising-Weihenstephan, Germany; 2 National University of Mongolia, Ikh surguulin gudalmj –1, Baga toiruu, Sukhbataar District, Ulaanbaatar, Mongolia; 3 State Key Laboratory of Vegetation and Environmental Change, Institute of Botany, Chinese Academy of Sciences, Beijing, China; The Pennsylvania State University, United States of America

## Abstract

We tested whether the abundance of C_4_ vegetation in grasslands of the Mongolian plateau is influenced by grazing conditions. The analysis exploited the politically originated contrast that exists between Mongolia (low stocking rate, transhumant system) and the district of Inner Mongolia, China (high stocking rate, sedentary system). We estimated the proportion of C_4_ carbon (P_C4_) in grazed vegetation from the relative carbon isotope ratio (δ^13^C) of sheep wool sampled from 298 annual shearings originating from 1996 to 2007. Annual stocking rates varying over time and between the districts of both countries were taken from regional statistics. The P_C4_ pattern within the 0.7 million km^2^ sampling area was geostatistically analyzed and related to stocking rates and temperature gradients. For similar climatic conditions, P_C4_ was the same in both countries. Further, a unique relationship was found between P_C4_ and July temperature on both sides of the border, which explained 71% of the pattern. Stocking rate and grazing system had no significant influences on present-day C_3_/C_4_ abundance ratio. This finding suggests that recent changes in the C_3_/C_4_ ratio of these grasslands are mainly a consequence of regional warming, not overgrazing.

## Introduction

The present-day distribution of C_4_ plants in grasslands worldwide is associated positively with temperature [1], and the grasslands of the Mongolian plateau largely fit into this pattern [2], [3]. This basic relationship is also known to be influenced additionally by other factors. Aridity, for instance, favors C_4_ vegetation in cool-temperate areas, and pH and salinity also show strong (if local) effects on C_4_ abundance ([1] and references therein). Conversely, the impact of grazing management on the C_3_/C_4_ composition of grasslands is less well documented.

By reducing vegetation cover and transpiration, grazing may lead to more sensible heat on the ground surface and thus to higher temperatures [4], thereby favoring C_4_ vegetation. Also, increases in stocking rate typically widen the gap between forage growth in early spring and the forage demand of livestock, with potentially the greatest damage to those plant species that are growing and consumed during this period, which are C_3_ species [5]. This may also reduce water use in spring and increase soil moisture in summer. In turn, more soil moisture during summer would favor C_4_ vegetation.

Understanding grazing effects is crucial, for example when modeling global C_4_ distribution and its impact on carbon cycling (e.g. [6], [7]). Although few studies have assessed grazing effects on the C_3_/C_4_ composition of grasslands, available results suggest that higher grazing pressure would favor C_4_ vegetation in semiarid climates (*steppes* of east Asia: [8]; *prairies* of the USA: [9], [10]). However, this effect is less evident in more humid grasslands [11], [10]. Furthermore, in a grazing experiment conducted on grassland in Inner Mongolia in which there was varying grazing pressure, no effect of grazing could be found [12]. This may have been due to the fact that the experiment was relatively short (six years) and did not include the winter grazing that is a typical feature of central Asia grassland. Whereas plants may adapt to high grazing pressure during the growing season this is not possible during the dormant season, and grazing may result in damage during that time. Furthermore, the conduct of grazing experiments requires the use of small fenced plots that differ in their management from the transhumant grazing system. Transhumance has been typical for central Asian grasslands for many centuries and is still in use in Mongolia while this has been replaced by a sedentary system in Inner Mongolia. Transhumance denotes a restricted form of nomadism where movement occurs in a largely fixed pattern between seasonally differing pastures in contrast to nomadic pastoralism with irregular, mostly long-distances movements to escape long-term feed shortage, e.g. due to droughts [13].

Long-term effects and management effects in which the management does not follow a strict experimental protocol but reacts to seasonally and annually changing grazing conditions can only be analyzed in field studies. Here, we test if the C_3_/C_4_ abundance ratio in grasslands of the Mongolian plateau is affected by stocking rate and grazing system (sedentary *vs.* transhumant). To do this, we take advantage of the existence of contrasting grazing management systems on different sides of the border between Inner Mongolia in China (high stocking rate, sedentary system) and Mongolia (low stocking rate, transhumant system) and the large contrast between years on both sides of the border (with annually varying stocking rates). The large contrast between the two countries in their grazing conditions is predicted to produce a discontinuity along the common border while the influence of gradually changing environmental conditions like climate would be expected to result in gradients of C_4_ abundance. To map and capture both, the discontinuity and possible gradients, distances of several hundred kilometer are necessary, which can be covered by making use of the “sampling” activity of the sheep that graze these grasslands. Our previous studies have shown that in these grasslands the δ^13^C of sheep wool can be interpreted in terms of the C_3_/C_4_ ratio of herbage on the grazing grounds of a flock [3], [14]. Wool from annual shearings integrates the C_3_/C_4_ signal of consumed biomass over one year and several square kilometers, thus accounting for the potentially high spatio-temporal variations caused by both local climatic/edaphic variation and by the asynchronous development of C_3_ and C_4_ plants. Due to this spatio-temporal integration the variance of δ^13^C in wool is half as large as that of the vegetation for the Inner Mongolia grassland [3] reducing the scatter and improving the detection of gradients. Further, wool captures the asynchronous growth of C_3_ and C_4_ plants even without repeated measurements at identical locations, which are possible in local experiments [12], [14] but cannot be achieved in a regional study covering an area of thousands of km^2^.

## Materials and Methods

### Land-use History

The Inner Mongolia Autonomous Region of China shares a 4700 km common border with Mongolia (formerly known as Republic of Mongolia) with very similar environmental conditions on both sides. The two countries were separated strictly as belonging to different political blocks during the Cold War. This separation brought about a large spatial contrast in land-use conditions. These differences still exist although both countries have opened and intensified their economies. The grazing conditions and stocking rates have changed as a result of these economic changes, but there is also considerable temporal variation that is typically caused by fluctuations in weather/growing conditions, especially due to winters with extraordinarily thick snow cover. During these periods many animals die because the thick snow cover prevents winter grazing and nomads cannot afford to conserve, transport or purchase additional feed. Historically, the entire grassland on the Mongolian plateau was used by transhumant pastoralists, who lived in gers (traditional Mongolian tents) and moved with their livestock to different seasonal pastures. In the case of extended feed shortage, they also moved over larger distances. In the late 1950s the herders in Inner Mongolia were forced to settle down [15] and live in farm-houses, and this has restricted their ability to respond to local droughts. Of much greater consequence, the number of sheep and goats increased from about 6 million to almost 90 million [16], greatly increasing grazing pressure. In consequence spring grazing rest periods (prohibition of grazing for 30–45 days between mid April and early June) have been enforced since 2001, although during most of the winter the system of winter grazing is still maintained.

In contrast, the nomadic system of livestock management endured in Mongolia even after the country became communist in 1924. Private property was re-established in 1990, and now nearly all livestock are owned by private households, most of which have retained their transhumant lifestyle [17]. The number of sheep and goats increased relatively moderately from about 17 million (1950) to about 36 million heads (2007) [18], [19]. During winter time, animals do not receive any supplementary fodder but stay at winter-grazing pastures [17].

### Study Area, Vegetation Structure, and Sampling Methods

**Figure 1:**
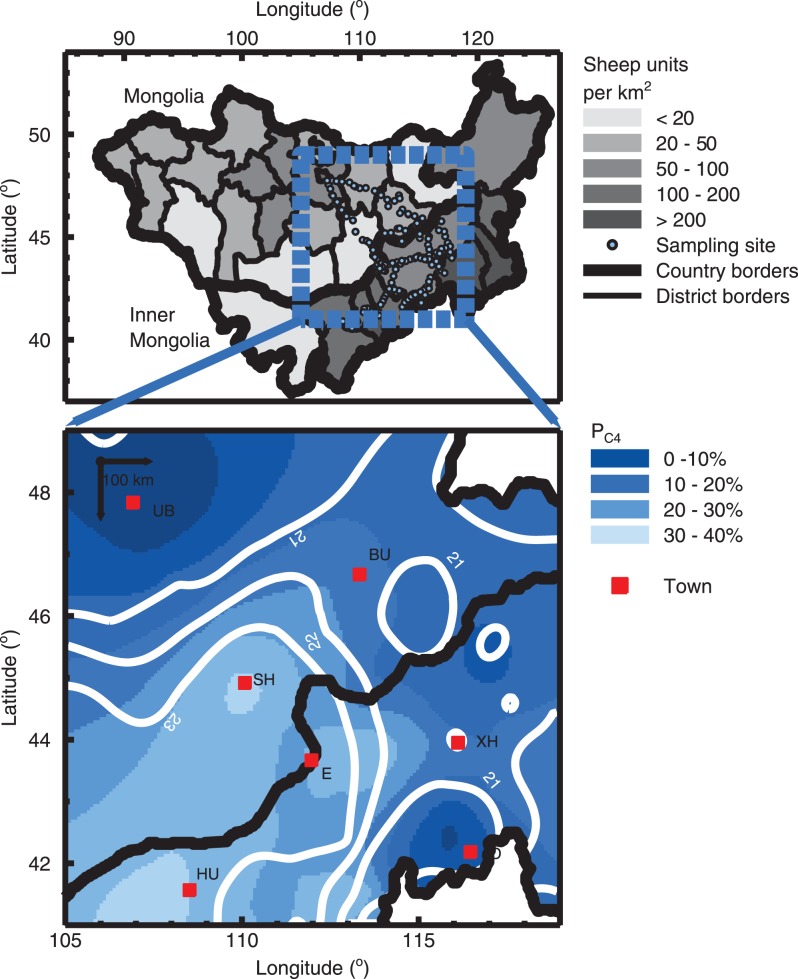
Sampling sites, stocking rates, proportion of C_4_ plants, and isotherms across Inner Mongolia and Mongolia. Top panel: Distribution of sampling sites and stocking rates across Inner Mongolia and Mongolia. Stocking rates were averaged for the years 1996 to 2007 and expressed as sheep units per km^2^ and yr for the individual districts. Bottom panel: Regional relative proportion of C_4_ plants to aboveground biomass (P_C4_) estimated by ordinary block (5×5 km^2^) kriging, derived from wool originating from 1996–2007; mean SD_k_ for the blocks is 8.9%; the white lines denote the 21, 22 and 23°C isotherms of the July temperature, averaged for the years 1996–2007; towns are UB = Ulaanbaatar, SH = Sainshand, E = Erenhot and Zamny-Uud, XH = Xilinhot, D = Duolun and HU = Haliut.

The study area was situated between 106°28′ and 117°49′E (approximately 850 km) and 41°34′ and 47°50′N (approximately 800 km) in Inner Mongolia and in Mongolia (Fig. 1 top). Sampled altitudes ranged from 800 m to 2000 m above sea level. Mean annual precipitation (MAP, mm yr^−1^) increases from less than 100 mm yr^−1^ in the Gobi Desert to more than 400 mm yr^−1^ in the eastern and northern parts of the study area. Approximately 75% of the precipitation falls during the vegetation growing period (April-September). Mean annual temperature and mean temperature of the growing period vary between −4 to 6°C and between 14 to 19°C, respectively.

The Inner Mongolia and Mongolia grasslands, representative of the Eurasian steppe region, are similar in terms of their vegetation structure, plant species composition, and evolutionary history of grazing [20], [21], [22], [23]. Along an east-west precipitation gradient, the study area includes four vegetation types: meadow steppe, typical steppe, desert steppe, and desert. The meadow steppe, located at the eastern part of the region, is dominated by *Stipa baicalensis* Roshev., *Leymus chinensis* (Trin.) Tzvel., and *Carex pediformis* C. A. Mey.; among the four vegetation types this steppe is highest in both species richness and aboveground biomass. The typical steppe is dominated by *Stipa grandis* P. Smirn., *L. chinensis, Stipa krylovii* Roshev., *Caragana microphylla* Lam., and *Cleistogenes squarrosa* (Trin.) Keng, with an intermediate level of productivity and plant species richness. The desert steppe at the western part of the region is dominated by *Stipa klemenzii* Roshev., *Allium polyrhizum* Turcz. ex Regel, and *Cleistogenes songorica* (Roshev.) Ohwi, with low species richness and productivity. At the western edge of the gradient is desert, which is dominated by xerophytic shrubs and annuals, including *Reaumuria soongorica* (Pall.) Maxim., *Salsola passerine* Bunge, *Nitraria sphaerocarpa* Maxim., and *Ceratoides lateens* (J. F. Gmel.) Reveal et Holmgren. At the cooler and wetter northern edge of the study area near Ulaanbaatar a fifth vegetation type can be found, the forest steppe, which forms a transition to the taiga that extends north of Ulaanbaatar. Typical species include *Agropyron cristatum* (L.) Gaertn., *S. krylovii*, *Carex duriuscula* C. A. Mey. and *Artemisia frigida* Willd. [20], [21], [22], [23].

The samples comprised almost entirely bulk wool from previous shearings (either raw wool from the last shearing or woolen artifacts like tent felt or mattresses) for which the year of shearing was known by the pastoralist. Hence, the wool reflected the vegetation eaten by the flock in the year previous to the shearing of the flock on the entire grazing area including seasonally changing places and thus leveling out of small-distance variations. Further, this sampling approach allowed us to sample several years simultaneously and thus to cover an identical time period across the entire region although the entire region could not be sampled within an individual year and in all years. The period average also levels out the effects of the strong fluctuations in growing conditions between individual years [12].

In Mongolia, 152 samples, dating from 1996–2006 were sampled on 65 sites in 2006. Transhumant movements in Mongolia were reconstructed from herders’ information. The maximum distance between summer and winter places was about 70 km, with an arithmetic mean of about 16 km and a geometric mean of about 8 km, and hence, the samples could be assigned relatively precisely to the location of sampling. Where long-distance movement had occurred (e.g. in response to dry conditions), the samples were assigned to the place of origin. Due to the on-going transhumant system, in which winter grazing occurs at different places than the summer grazing, no winter feed is conserved and winter feeding is entirely based on grazing.

In Inner Mongolia samples were collected in 2003, 2004, 2005, 2007 and 2009. Isotopic data from the 2004 to 2007 collections were previously reported by [3] together with a comparison to the vegetation samples. Overall, 146 wool samples dating from 1998–2009 from 101 sites in Inner Mongolia were used.

The constraint of the grazing rest practiced since 2001 in Inner Mongolia was partially compensated by the distribution of 0.15 kg d^−1^ animal^−1^ of concentrate pellets, which consisted mainly of maize (on average 65%). The main pen fodder supplied during the rest time was still hay produced by the herders on their local (grazed) pastures. Overall, the pellets added about 1% C_4_ to the annual diet of sheep and goats (for calculation see Table S1). Animals did not receive supplements on pasture. Regular pen fodder in the winter and/or spring consisted of hay from local meadows and meadow steppes (information from local herders).

All necessary permits were obtained for the field studies described. In cases where the land was not public but assigned to a pastoralist we had his permission to enter his land because he provided us with the wool samples and the information about their ages and land-use related data. In the case of publicly owned land in the border region we had a permit issued by the Border Military Department of Mongolia. The study did not involve sampling of any protected area or of any protected or endangered species.

### Sample Preparation, Isotope Analysis and Estimation of C_4_ Fraction

Sample preparation, isotope analysis and estimation of the C_4_ fraction were identical to the procedures described in [3]. In brief, the wool was cleaned, cut into small pieces and 0.2 to 0.4 mg were packed into tin cups for isotope analysis. The carbon isotope composition of the samples (δ^13^C) was determined by isotope ratio mass spectrometry with a precision of sample repeats (standard deviation, SD) better than 0.1‰.

The proportion of C_4_ plants (P_C4_) in aboveground biomass at each site was estimated from δ^13^C of the wool samples according to:
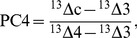
(1)with ^13^Δ_c_ denoting whole-community carbon isotope discrimination and ^13^Δ_3_ and ^13^Δ_4_ the discrimination of the local C_3_ and C_4_ end-members of the mixing model. Discrimination ^13^Δ between the atmospheric CO_2_ source (average of growing season of respective years) and the product (C_3_ or C_4_ vegetation) is given by
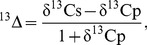
(2)where the subscript s denotes the source and p the product. 13Δc was calculated as 13Δwool +3.2‰, the constant fractionation between vegetation and wool [14]. Site-specific 13Δ3 was estimated from year-specific growing-season precipitation, PG in mm d−1, according to [24] and altitude, A in m above sea level, according to [25] as
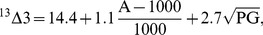
(3)and 13Δ4 was taken as a constant 7.2‰ from [12]. The relatively high 13Δ4 is mainly caused by C. squarrosa, which dominates the C4 community and which has a high leakiness affecting 13Δ [26]. A bias due to the selection of sheep or differences in the digestibility of C3 and C4 components was unlikely over the entire range of grazing intensities, as was confirmed by the results from a controlled grazing experiment [14], [27]. Also, a change in species composition within the C3 and C4 communities and a change in species discrimination as a response to varied grazing intensities caused 13Δ3 and 13Δ4 to deviate by not more than 0.2‰ [12] while the difference between both endmembers is about 10‰. Hence the endmembers can be estimated without knowledge of the grazing intensity.

### Meteorological, Livestock and Geographical Data

Coordinates and altitude of the sampling sites were measured with a global positioning system. Site- and year-specific precipitation and temperature data were obtained following [14] by correcting normal-period high-resolution (2 km×2 km) maps for the deviation of an individual year, which was geostatistically interpolated from daily precipitation and temperature data of 63 climate stations (http://cdo.ncdc.noaa.gov/CDO/cdo). For statistical analysis, we used either the temperature in July for the sampling location and year of each sample (T_Jul_) or the average for the observation period 1996 to 2007 (T_Jul 96–07_).

Livestock data and land area of administrative units (called League in Inner Mongolia and Aimag in Mongolia) were taken from the official statistical yearbooks [19], [16]. Livestock data were converted to sheep units (SU) according to Table S2. Borders between countries and districts (Leagues, Aimags) were taken from GADM (www.gadm.org).

P_C4_ was related to the stocking rate of the respective years and districts. Thus, stocking rates varied between years on identical sites and they varied between sites. This two-fold variation created a large range and avoided collinearity.

### Statistical and Geostatistical Analysis

Linear regressions and multiple linear regressions were used to evaluate the data. The coefficient of determination was tested with a two-sided test for significance at thresholds of *P*<0.05, *P*<0.01, and *P*<0.001. Statistical spread is denoted as 95% confidence interval of the mean. To test if the level of P_C4_ differed between Inner Mongolia and Mongolia we used Student’s t-test on parity of the means of two populations (two-sided), preceded by an F-test on equality of variances. Sub-populations were selected by restricting the data to the sampling points within a 100 km belt to each side along the common border. We used only data that originated from the overlapping period of 1998 to 2006 in order to exclude bias caused by inter-annual fluctuations. All statistical procedures were performed with R 2.9.1 [28] following standard protocols.

Geostatistics quantify the nature of spatial dependence of a given property (e.g. P_C4_). This allows separating the data uncertainty from the spatial pattern, to interpret the pattern, and to estimate the property at unrecorded positions (see [29] and citations therein). The semivariance equals the variance of a property at points, which are separated by a certain distance called lag. The semivariances for classes of lags yield the empirical semivariogram. A theoretical semivariogram was fitted to minimize weighted least squares. The theoretical semivariogram delivers three parameters: the nugget effect, the sill, and the range. The nugget effect quantifies the small-scale variation including data uncertainty. The sill quantifies the total variation caused by the nugget effect and the variation due to a spatial pattern. The nugget/sill ratio hence reflects the ratio of random-(unexplained by a pattern)-to-total variation. The range quantifies the distance of autocorrelation caused by the pattern.

Maps were constructed for a uniform rectangular grid by ordinary block kriging (for 27000 5×5 km^2^ grid cells) to remove the noise and retrieve the pattern by using the theoretical semivariogram and all measured data. The quality of the predictions from the resulting maps was given as the (block) krige standard deviation (SD_k_), which is a measure of the prediction error of an individual block. Data handling and geostatistical and spatial analysis were also carried out with R 2.9.1 using the auxiliary packages gstat [30] and maptools [31].

## Results

### Stocking Rates in Inner Mongolia and Mongolia

**Figure 2:**
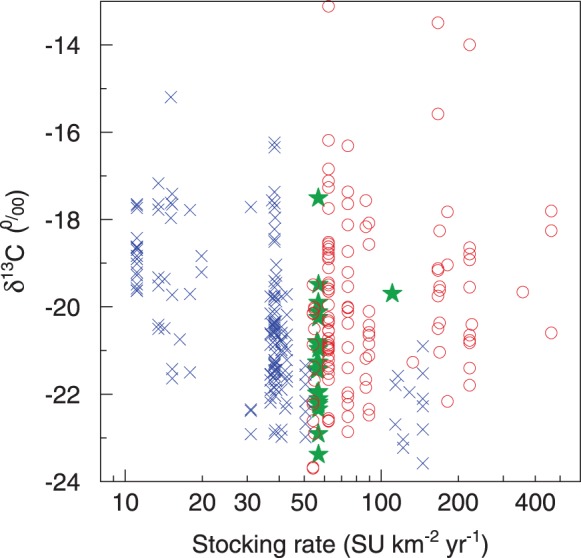
Carbon isotope ratio does not depend on stocking rate. Relative carbon isotope ratio (δ^13^C) in relation to stocking rate (log-scaled to increase readability) in different districts and years in Mongolia (blue crosses, all years; n = 152) and in Inner Mongolia prior to 2001 (green stars; n = 22) and after 2001 (red circles; n = 124).

Small ruminants (sheep and goats) accounted for 55% of the total livestock numbers in Inner Mongolia and 43% in Mongolia. The remainder was mainly cattle and horses, which were small in number but contributed together about 45% of the sheep units. The contribution of horses was larger in Mongolia (20% *vs*. 4%) where horses are still valued due the continued existence of transhumant grazing. The range of stocking rates was large in both regions: it varied from 54 to 462 SU km^−2^ yr^−1^ in Inner Mongolia and from 11 to 145 SU km^−2^ yr^−1^ in Mongolia (Fig. 2) between districts and years. The average stocking rate in the years 1996 to 2007 was about twice as high in Inner Mongolia (82 SU km^−2^ yr^−1^) as it was in Mongolia (40 SU km^−2^ yr^−1^). Within the 100 km-wide zone along the common border, stocking rates were on average even three times higher in Inner Mongolia than in Mongolia (Table 1). This larger contrast along the border than between the country averages was because in Mongolia the stocking rates were highest near to Ulaanbaatar where population density also peaked, and then they decreased in the direction of the border to the south.

**Table 1:** Comparison of 100 km-wide zones along the common border of Inner Mongolia and Mongolia regarding the number of samples (N), the stocking rate for the years of wool growth (expressed as sheep units SU), the proportion of C4 (P_C4_), the mean annual precipitation (MAP), the mean July temperature for the years of wool growth (T_Jul_), and the respective 95% confidence intervals of the means (CI_95%_).

		Inner Mongolia	Mongolia
N		38	67
Stocking rate (SU km^−2^ yr^−1^)	Mean¶	67.7^A^	24.1^B^
	CI_95%_	2.6	3.0
P_C4_ (%)	Mean¶	17.5^C^	21.9^C^
	CI_95%_	4.2	3.1
MAP (mm yr^−1^)	Mean¶	196^D^	175^E^
	CI_95%_	16	12
T_Jul_ (°C)	Mean¶	22.7^F^	22.7^F^
	CI_95%_	0.5	0.5

¶ Different capital letters within a line denote significant differences (*P*<0.05) between means of Inner Mongolia and Mongolia.

### P_C4_ and Environmental Conditions in Inner Mongolia and Mongolia

Wool originating from prior to the rest of spring grazing (i.e., before 2001) in Inner Mongolia exhibited no distinctly higher δ^13^C than samples originating after the prohibition of spring grazing (Fig. 2; *P*>0.5). No influence of stocking rate on δ^13^C was apparent for the entire data set (Fig. 2). The same was true when δ^13^C was converted to P_C4_. Within the Mongolia samples there was a significant decrease in P_C4_ with increasing stocking rate, which was caused by the collinearity of temperature and stocking rate. Approaching Ulaanbaatar, the most northern sampling location, T_Jul_ decreased from 23°C near the border to 20°C while stocking rates increased from 11 to 145 SU km^−2^. Dividing the entire data set into three (almost evenly occupied) July temperature classes, the stocking rate had no influence but mean P_C4_ significantly increased from 12.4±2.8% (for T_Jul_≤20.5°C), to 18.0±3.1% (for 20.5°C<T_Jul_ ≤22.5°C), to 21.8±3.1% (for T_Jul_>22.5°C).

Restricting the data to the 100 km-wide border zone with near-identical conditions on both sides, 38 wool samples from 21 farms originated from Inner Mongolia and 67 samples from 31 herders came from Mongolia. The means of the respective P_C4_ sample populations were not significantly different (*P*>0.05, Table 1) despite a highly significant difference of approximately 44 SU km^−2^ yr^−1^ between the sampling points in Inner Mongolia and those in Mongolia. T_Jul_ exhibited no difference between both countries in the border zone while MAP was slightly lower in Mongolia, owing to the more continental climate, than in Inner Mongolia (Table 1).

### Geographical Variation of P_C4_, Temperature and Stocking Rate

C4 vegetation accounted for a maximum of 85% of an individual sample reflecting the aboveground biomass at the scale of grazing grounds of individual herds, and for 18±2% (CI) on average. In multiple linear regressions taking into account T_Jul_, stocking rate exhibited no influence on P_C4_ despite the large variation (42-fold) and sample size (n = 298, Fig. 2, for the regression equations see Table S3, for the complete data set see Table S4). In contrast T_Jul_ was always highly significantly related to P_C4_ (Table S3).

**Figure 3:**
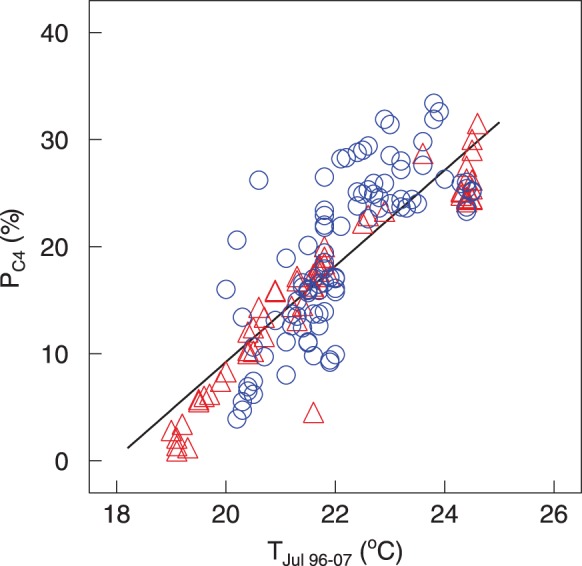
Contribution of C_4_ plants to aboveground biomass increases with July temperature. Relative contribution of C_4_ plants to aboveground biomass after geostatistical averaging (kriged P_C4_) at the sampling sites (n = 166) in relation to the mean July temperature, averaged for the years 1996–2007 (T_Jul, 96–07_); red triangles denote sites in Inner Mongolia and blue circles denote those in Mongolia; the line is the linear regression (r^2^ = 0.71).

After removal of small-scale uncertainty *via* kriging, P_C4_ ranged from 1% to 34% with a mean of 18%. Kriged P_C4_ increased by 4.5% per 1°C increase in T_Jul__96–07_. T_Jul__96–07_ explained 71% of the regional scale variation in P_C4_ obtained by kriging (*P*<<0.001; Fig. 3). Indeed, the spatial pattern of P_C4_ agreed well with isotherms of T_Jul__96–07_ (Fig. 1 bottom), which delineated areas of high P_C4_ (in the south-west, i.e. the Gobi desert) from areas of low P_C4_ (in the north and south-east).

Within the study area, growing period precipitation and T_Jul_ were negatively correlated (for individual years during the sampling period r^2^ = 0.37; for the means during the last normal period r^2^ = 0.75). In the drier and warmer area of the Gobi desert sufficient rainfall occurs only during the warmest months (June, July and August), whereas in the cooler areas spring and autumn months also receive sufficient rain to sustain growth. Hence, differences in temperature for the period with sufficient precipitation are larger than differences in T_Jul_.

## Discussion

### (1) Effect of Summer Temperature on P_C4_

Our findings demonstrated a large plasticity in P_C4_ ranging up to 84% in individual samples. This plasticity between years at individual locations has also been found previously in an experiment with small plots (2 ha) and fixed stocking rates [12]. Despite this interannual plasticity, a clear regional pattern in P_C4_ was obtained that agreed with the pattern of T_Jul_ (either when comparing individual years or 10-yr averages). Two reasons may contribute to this large effect:

First, the area is close to the estimated crossover temperature where a change in temperature should have the largest impact. The crossover temperature is given as the point at which the lines of net photosynthesis *versus* temperature of C_3_ and C_4_ species intersect [7]. For the CO_2_ concentration during the growing period of the study (1996–2007), crossover temperature is approximately 22°C for a light saturated stand [7]. For the observed total range in T_Jul_ in individual years (18°C–27°C) an increase in net photosynthesis of approximately 120% can be expected for C4 species, while net photosynthesis only increases by 15% for C_3_ species following [7] when assuming a volumetric CO_2_ concentration in the atmosphere of 350 ppm, a leaf temperature equal to air temperature, and light saturated conditions in these grasslands where vegetation cover rarely exceeds 50%.

Second, other temperature-related processes than net photosynthesis that are not captured by the crossover temperature may also contribute to the large effect of temperature on the regional pattern. These include: (i) germination temperature, which follows the temperature preference of a plant [32], may contribute, as all C_4_ species in the study area can be classified as ruderals and one of the two dominating C_4_ species, *Salsola collina* Pall., is an annual species. (ii) With increasing T_Jul_ the favorable temperatures for C_4_ growth are reached earlier in the season and last longer, and hence the period favorable for C_4_ growth increases in length relative to the period of growth for C_3_ species (data not shown). (iii) Aridity, which promotes C_4_ species [1], [2], increases due to the negative correlation of temperature with precipitation and its positive correlation with evaporation. (iv) High temperatures tend to decrease the leakiness (i.e. the fraction of CO_2_ generated by C_4_ acid decarboxylation that subsequently leaks from bundle-sheath cells) in C_4_ grasses [33] that reduces the light use efficiency. The most abundant C_4_ species, *C. squarrosa,* exhibits a large ^13^C discrimination caused by a high leakiness [26], and this species may therefore additionally profit from high temperatures.

### (2) Effects of Grazing Regime and Stocking Rate on P_C4_

Stocking rates during the study period were two to three times greater in Inner Mongolia than in Mongolia. Further, compared to the transhumant grazing system prevalent in Mongolia region, sedentary grazing (*i.e.* a fixed stocking rate) would have amplified the effects of the higher stocking rates in Inner Mongolia. But despite such strong differences in grazing conditions, no evidence was observed for major grazing effects on the C_3_/C_4_ composition of the semi-arid grasslands across the Mongolian plateau. P_C4_ was 18–21% in both countries, no relationship was observed with stocking rate in areas of low, medium or high temperature, and no substantial difference was observed in the relationship between P_C4_ and T_Jul_ for areas with different stocking rates neither between nor within countries. This corroborates the results from a local grazing experiment [12] and further excludes the likelihood that grazing regime or long-term effects create a response.

This lack of an effect is likely due to the fact that the dominating C_4_ species (*C. squarrosa, S. collina*) are grazing tolerant and hence could be found independently of grazing pressure. However, neither of these species profited from high grazing pressure, which is contrary to the hypothesis in which it had been predicted that: (i) the expected changes in microclimate would favor C_4_ species, (ii) selective grazing damages C_3_ species in early spring and (iii) improves the water availability of C_4_ species. The hypothetical mechanisms must therefore have been counteracted by other mechanisms:

- A grazing-induced (local) change in microclimate would necessarily have scaled up to the macroclimate, due to the large-scale difference in grazing intensity on the Mongolian plateau with the research area covering about 0.7 million km^2^. Temperature isotherms, however, showed no mismatch or displacement along the Mongolian border during the study period (Fig. 2). Measurements have shown that the albedo of bare soil under dry conditions is higher than that of vegetation covered surfaces [4]. This leaves less energy for heating, even if biomass removal lowers evapotranspiration (see discussion by [4]).
- The reasons why grazing does not selectively damage C_3_ species in early spring are less clear. First, all regional species are adapted to (normal) grazing and severe drought and can survive with little growth over several years. Second, many C_3_ species can partly survive the effects of light grazing in spring by adapting their growth habit, whereas a period of intensive grazing pressure, during which nearly all aboveground biomass is removed, would also cause feed shortage later in the season and thus damage the C_4_ species at the stage when they are starting to produce leaves.
- The competitive advantage of C_4_, caused by a better access to soil moisture after C_3_ removal, presumably is small. To have such an advantage, the dominant C_4_ species (*C. squarrosa* and *S. collina*) must access the soil water that is normally utilized by C_3_ species. The dominant members of the C_3_ community (*S. grandis* and *L. chinensis*), however, compete only partly with the C_4_ species for topsoil water, but additionally they can access subsoil water [34]. To profit from the removal of C_3_ species, the C_4_ species would need a deep root system, which is neither the case for the perennial *C. squarrosa* nor the annual *S. collina* [34], [35].

However, despite the apparent lack of an influence of grazing intensity on the C_3_/C_4_ ratio, de-stocking is urgently needed on the Mongolian plateau as a consequence of the more than 10-fold increase in stocking rates that have occurred during the past half century and the simultaneously ongoing climate change and thus C_4_ expansion [36]. Overgrazing, which decreases plant diversity and productivity [23], may further diminish ecosystem resilience to climatic warming and prolonged droughts in this region [37]. The lower C_3_ abundance lowers primary productivity in early spring, which in turn reduces the carrying capacity and facilitates grassland degradation.

### Conclusions

Stocking rate was two- to threefold higher in Inner Mongolia (China) than in Mongolia. In addition, sedentary grazing in Inner Mongolia prevented herders from adjusting the grazing pressure in response to annual and regional variation in precipitation. Despite this large contrast we found no evidence of influence of grazing on the contribution of C_4_ vegetation to annual production. The spatial pattern of C_4_ contribution in both regions was predominantly associated with T_Jul_, the temperature during the warmest month. The recent expansion of C_4_ vegetation in this ecosystem [36] seems not to be driven by grazing effects. Regional warming is a more likely cause. This lack of grazing effects on the C_3_/C_4_ composition does not, however, mean that overgrazing is not occurring. De-stocking is urgently needed on the Mongolian plateau because of diminishing soil cover, increased erosion, and decreasing contribution of valuable forage species.

## Supporting Information

Table S1 Calculation of the C_4_ proportion in annual feed intake provided by pellets
(DOC)

Table S2 Livestock equivalents, expressed as sheep units (SU), of different types of livestock in the grassland on the Mongolian plateau
(DOC)

Table S3 Linear (1, 2, 4, 5) and multiple (3) regression parameters for regressions of the form y = β_0_+x_1_×β_1_+x_2_×β_2_ with y denoting P_C4_ and x_1_ and x_2_ denoting either July temperature (T_Jul_) or stocking rate (expressed as SU km^−2^ yr^−1^) or a combination of both for the respective year from which the sample originated (n = 298). The parameters (SU × T_Jul_) and (SU/T_Jul_) were examined to identify possible interactions.
(DOC)

Table S4 Location of sampling, country (IM = Inner Mongolia, MN = Mongolia), year before shearing ( = year of biomass growth), stocking rate in the year before shearing in the respective district (aimag or league) in sheep units per km^2^ and year, temperature in July (T_Jul_ in °C) and carbon isotopic composition of wool (δ^13^C in ‰).
(DOC)
