# Supplementary material for: Sheep Wool δ13C Reveals No Effect of Grazing on the C3/C4 Ratio of Vegetation in the Inner Mongolia–Mongolia Border Region Grasslands
Source: PLoS One. 2012 Sep 27;7(9):e45552. doi: 10.1371/journal.pone.0045552 (PMC3459995; doi:10.1371/journal.pone.0045552)
Supplement: Table S1 — Calculation of the C4 proportion in annual feed intake provided by pellets (DOC) [file pone.0045552.s001.doc]

**Table A1** Calculation of the C4 proportion in annual feed intake provided by pellets

|  | **Amount and unit** | **Remark** |
| --- | --- | --- |
| A Daily compensation: | 0.15 kg d^-1^ SU^-1^ |  |
| B Duration: | 30-45 d yr^-1^ |  |
| C Total compensation: | 4.5-6.75 kg yr^-1^ SU^-1^ | C = A × B |
| D C4 proportion in compensation: | 65% | measured |
| E Total C4 in compensation: | 2.9-4.4 kg yr^-1^ SU^-1^ | E = C × D |
| F Daily organic matter intake: | 1.2 kg d^-1^ SU^-1^ | [36], valid for summer grazing period |
| G Annual organic matter intake: | 1 kg d^-1^ SU^-1^ | Assumed from F and a lower intake during winter, when animals loose weight |
| H Total feed intake: | 365 kg yr^-1^ SU^-1^ | H = G × (365 d yr^-1^) |
| I Additional C4: | 1% | I = E / H × 100% |

36. Glindemann T, Wang C, Tas BM, Schiborra A, Gierus M, Taube F, Susenbeth A (2009) Impact of grazing intensity on herbage intake, composition, and digestibility and on live weight gain of sheep on the Inner Mongolian steppe. Livestock Science 124: 142-147.
