# Supplementary material for: Sheep Wool δ13C Reveals No Effect of Grazing on the C3/C4 Ratio of Vegetation in the Inner Mongolia–Mongolia Border Region Grasslands
Source: PLoS One. 2012 Sep 27;7(9):e45552. doi: 10.1371/journal.pone.0045552 (PMC3459995; doi:10.1371/journal.pone.0045552)
Supplement: Table S2 — Livestock equivalents, expressed as sheep units (SU), of different types of livestock in the grassland on the Mongolian plateau (DOC) [file pone.0045552.s002.doc]

**Table A2** Livestock equivalents, expressed as sheep units (SU), of different types of livestock in the grassland on the Mongolian plateau

| **Animal** | **Sheep unit (SU)** |
| --- | --- |
| Sheep | 1 |
| Goat | 0.9 |
| Cattle, horse, donkey or mule | 6 |
| Camel | 5.4 |
