# Supplementary material for: Sheep Wool δ13C Reveals No Effect of Grazing on the C3/C4 Ratio of Vegetation in the Inner Mongolia–Mongolia Border Region Grasslands
Source: PLoS One. 2012 Sep 27;7(9):e45552. doi: 10.1371/journal.pone.0045552 (PMC3459995; doi:10.1371/journal.pone.0045552)
Supplement: Table S3 — Linear (1, 2, 4, 5) and multiple (3) regression parameters for regressions of the form y = β0+x1×β1+x2×β2 with y denoting PC4 and x1 and x2 denoting either July temperature (TJul) or stocking rate (expressed as SU km−2 yr−1) or a combination of both for the respective year from which the sample originated (n = 298). The parameters (SU × TJul) and (SU/TJul) were examined to identify possible interactions. (DOC) [file pone.0045552.s003.doc]

**Table A3** Linear (1, 2, 4, 5) and multiple (3) regression parameters for regressions of the form y=β_0_+x_1_×β_1_+x_2_×β_2_ with y denoting P_C4_ and x_1_ and x_2_ denoting either July temperature (T_Jul_) or stocking rate (expressed as SU km^-2^ yr^-1^) or a combination of both for the respective year from which the sample originated (n = 298); the parameters (SU × T_Jul_) and (SU / T_Jul_) were examined to identify possible interactions.

| **Model** | **β_0_** |  | **x_1_** | | |  | **x_2_** | | |  | **r^2^** |
| --- | --- | --- | --- | --- | --- | --- | --- | --- | --- | --- | --- |
|  |  |  | **Parameter** | **β_1_** | ***P*** |  | **Parameter** | **β_2_** | ***P*** |  |  |
| 1 | -26.40 |  | T_Jul_ | 2.02 | <<0.001 |  |  |  |  |  | 0.060 |
| 2 | 16.77 |  | SU | 0.01 | 0.358 |  |  |  |  |  | 0.003 |
| 3 | -28.95 |  | T_Jul_ | 2.08 | <<0.001 |  | SU | 0.02 | 0.18 |  | 0.060 |
| 4 | 16.46 |  | SU × T_Jul_ | 0.001 | 0.203 |  |  |  |  |  | 0.005 |
| 5 | 17.16 |  | SU / T_Jul_ | 0.16 | 0.613 |  |  |  |  |  | 0.001 |
