# Supplementary material for: Sheep Wool δ13C Reveals No Effect of Grazing on the C3/C4 Ratio of Vegetation in the Inner Mongolia–Mongolia Border Region Grasslands
Source: PLoS One. 2012 Sep 27;7(9):e45552. doi: 10.1371/journal.pone.0045552 (PMC3459995; doi:10.1371/journal.pone.0045552)
Supplement: Table S4 — Location of sampling, country (IM = Inner Mongolia, MN = Mongolia), year before shearing ( = year of biomass growth), stocking rate in the year before shearing in the respective district (aimag or league) in sheep units per km2 and year, temperature in July (TJul in °C) and carbon isotopic composition of wool (δ13C in ‰). (DOC) [file pone.0045552.s004.doc]

**Table A4** Location of sampling, country (IM = Inner Mongolia, MN = Mongolia), year before shearing (= year of biomass growth), stocking rate in the year before shearing in the respective district (aimag or league) in sheep units per km² and year, temperature in July (T_Jul_ in °C) and carbon isotopic composition of wool (δ^13^C in ‰).

| **Easting** | **Northing** | **Country** | **Year** | **Stocking rate** | **T_Jul_** | **δ^13^C** |
| --- | --- | --- | --- | --- | --- | --- |
| 115.880 | 42.830 | IM | 2003 | 62 | 19.3 | -22.62 |
| 115.550 | 42.180 | IM | 2003 | 62 | 19.4 | -20.96 |
| 116.000 | 43.030 | IM | 2003 | 62 | 19.5 | -21.53 |
| 114.711 | 42.187 | IM | 2006 | 226 | 19.7 | -20.40 |
| 115.100 | 45.150 | IM | 2003 | 62 | 20.0 | -21.36 |
| 115.220 | 41.800 | IM | 2003 | 62 | 20.0 | -22.56 |
| 115.000 | 44.080 | IM | 1998 | 56 | 20.1 | -21.98 |
| 115.100 | 44.950 | IM | 2003 | 62 | 20.1 | -20.84 |
| 116.870 | 43.380 | IM | 2003 | 182 | 20.1 | -22.17 |
| 117.250 | 45.030 | IM | 1998 | 56 | 20.1 | -21.27 |
| 117.470 | 44.720 | IM | 2004 | 74 | 20.1 | -20.93 |
| 116.700 | 43.530 | IM | 2003 | 62 | 20.1 | -20.89 |
| 116.120 | 43.650 | IM | 2003 | 62 | 20.1 | -18.86 |
| 116.680 | 43.570 | IM | 2003 | 62 | 20.2 | -20.55 |
| 116.680 | 43.580 | IM | 2003 | 62 | 20.2 | -19.59 |
| 116.720 | 43.570 | IM | 2003 | 62 | 20.2 | -13.12 |
| 116.720 | 43.570 | IM | 2003 | 62 | 20.2 | -19.96 |
| 116.720 | 43.580 | IM | 2003 | 62 | 20.2 | -22.61 |
| 116.730 | 43.580 | IM | 2003 | 62 | 20.2 | -20.93 |
| 116.730 | 43.580 | IM | 2003 | 62 | 20.2 | -20.54 |
| 115.880 | 45.020 | IM | 2003 | 62 | 20.2 | -21.23 |
| 115.830 | 44.920 | IM | 2003 | 62 | 20.2 | -21.13 |
| 116.600 | 43.780 | IM | 2003 | 62 | 20.2 | -20.99 |
| 116.500 | 43.820 | IM | 2003 | 62 | 20.2 | -19.45 |
| 117.670 | 44.550 | IM | 2003 | 62 | 20.2 | -21.41 |
| 117.200 | 43.250 | IM | 2003 | 182 | 20.3 | -19.04 |
| 117.280 | 44.470 | IM | 2003 | 62 | 20.3 | -19.59 |
| 116.500 | 44.250 | IM | 2003 | 62 | 20.3 | -20.32 |
| 116.930 | 44.430 | IM | 2003 | 62 | 20.3 | -21.67 |
| 115.800 | 44.620 | IM | 2003 | 62 | 20.3 | -20.72 |
| 115.430 | 44.000 | IM | 1998 | 56 | 20.3 | -21.45 |
| 115.870 | 42.500 | IM | 2002 | 55 | 20.3 | -21.38 |
| 115.900 | 44.400 | IM | 2003 | 62 | 20.3 | -18.90 |
| 115.900 | 43.930 | IM | 2003 | 62 | 20.3 | -16.84 |
| 115.900 | 44.130 | IM | 2003 | 62 | 20.4 | -18.78 |
| 115.520 | 43.920 | IM | 2003 | 62 | 20.4 | -20.36 |
| 116.780 | 44.200 | IM | 2004 | 74 | 20.5 | -22.23 |
| 117.680 | 44.270 | IM | 2003 | 62 | 20.6 | -20.25 |
| 115.180 | 43.970 | IM | 2003 | 62 | 20.6 | -16.19 |
| 117.250 | 45.030 | IM | 2004 | 74 | 20.7 | -21.97 |
| 116.430 | 43.880 | IM | 2004 | 74 | 20.7 | -20.01 |
| 116.930 | 45.770 | IM | 2003 | 62 | 20.7 | -20.94 |
| 114.800 | 44.020 | IM | 2003 | 62 | 20.8 | -19.11 |
| 114.280 | 43.920 | IM | 2003 | 62 | 20.8 | -17.27 |
| 114.114 | 41.956 | IM | 2007 | 222 | 20.9 | -21.40 |
| 113.818 | 41.820 | IM | 2007 | 222 | 21.0 | -20.65 |
| 113.850 | 43.830 | IM | 2003 | 62 | 21.0 | -18.62 |
| 115.850 | 42.632 | IM | 2007 | 90 | 21.0 | -22.48 |
| 117.820 | 43.980 | IM | 2003 | 182 | 21.0 | -17.83 |
| 117.170 | 45.270 | IM | 2004 | 74 | 21.0 | -22.52 |
| 116.720 | 45.330 | IM | 2004 | 74 | 21.1 | -20.02 |
| 115.631 | 42.548 | IM | 2007 | 90 | 21.1 | -21.12 |
| 114.367 | 42.099 | IM | 2007 | 222 | 21.1 | -20.82 |
| 115.421 | 42.422 | IM | 2007 | 90 | 21.1 | -20.85 |
| 113.607 | 41.595 | IM | 2007 | 222 | 21.2 | -14.00 |
| 115.165 | 42.331 | IM | 2007 | 90 | 21.2 | -22.35 |
| 114.711 | 42.187 | IM | 2007 | 222 | 21.2 | -21.80 |
| 117.470 | 44.720 | IM | 2002 | 55 | 21.3 | -20.50 |
| 115.430 | 44.000 | IM | 2004 | 74 | 21.3 | -17.37 |
| 113.050 | 43.750 | IM | 1998 | 56 | 21.3 | -21.42 |
| 113.470 | 43.700 | IM | 2003 | 62 | 21.4 | -17.12 |
| 116.930 | 45.770 | IM | 2004 | 74 | 21.4 | -22.86 |
| 114.711 | 42.187 | IM | 2001 | 133 | 21.5 | -21.27 |
| 115.830 | 44.920 | IM | 2002 | 55 | 21.5 | -21.68 |
| 115.000 | 44.080 | IM | 2004 | 74 | 21.6 | -16.31 |
| 116.680 | 43.570 | IM | 2002 | 55 | 21.7 | -20.05 |
| 113.170 | 43.780 | IM | 2003 | 62 | 21.7 | -17.75 |
| 115.720 | 43.900 | IM | 2005 | 87 | 21.7 | -21.85 |
| 115.870 | 43.950 | IM | 2005 | 87 | 21.8 | -21.66 |
| 111.689 | 41.307 | IM | 2004 | 358 | 21.8 | -19.66 |
| 113.050 | 43.750 | IM | 2003 | 62 | 21.8 | -19.50 |
| 113.050 | 43.430 | IM | 2003 | 62 | 21.8 | -18.68 |
| 110.375 | 41.512 | IM | 2007 | 169 | 21.8 | -18.26 |
| 116.500 | 44.250 | IM | 2002 | 55 | 21.9 | -22.90 |
| 113.900 | 43.830 | IM | 2004 | 74 | 21.9 | -18.73 |
| 114.670 | 43.970 | IM | 2005 | 87 | 22.0 | -21.18 |
| 112.870 | 43.100 | IM | 2003 | 62 | 22.0 | -20.84 |
| 110.321 | 41.344 | IM | 2007 | 169 | 22.0 | -19.54 |
| 110.321 | 41.344 | IM | 2007 | 169 | 22.0 | -20.37 |
| 113.706 | 42.301 | IM | 2007 | 90 | 22.0 | -20.66 |
| 110.651 | 41.529 | IM | 2007 | 169 | 22.0 | -19.62 |
| 112.700 | 42.780 | IM | 2003 | 62 | 22.1 | -19.65 |
| 116.680 | 43.570 | IM | 1999 | 57 | 22.2 | -23.38 |
| 116.720 | 43.570 | IM | 1999 | 57 | 22.2 | -22.11 |
| 113.570 | 44.800 | IM | 2002 | 55 | 22.2 | -20.50 |
| 115.430 | 44.000 | IM | 2002 | 55 | 22.2 | -20.01 |
| 113.580 | 43.850 | IM | 2004 | 74 | 22.3 | -20.38 |
| 113.542 | 42.340 | IM | 2007 | 90 | 22.4 | -20.59 |
| 110.929 | 41.386 | IM | 2007 | 169 | 22.4 | -21.04 |
| 112.470 | 43.680 | IM | 2003 | 62 | 22.4 | -19.65 |
| 115.880 | 45.020 | IM | 2001 | 54 | 22.5 | -19.50 |
| 112.150 | 43.350 | IM | 2003 | 62 | 22.6 | -18.56 |
| 111.765 | 41.707 | IM | 2007 | 222 | 22.6 | -18.65 |
| 115.900 | 43.930 | IM | 1999 | 57 | 22.6 | -20.96 |
| 111.970 | 43.380 | IM | 2003 | 62 | 22.7 | -18.52 |
| 111.689 | 41.307 | IM | 2007 | 462 | 22.7 | -18.26 |
| 111.920 | 43.530 | IM | 1998 | 56 | 22.7 | -20.80 |
| 112.500 | 42.480 | IM | 2004 | 74 | 22.7 | -19.54 |
| 111.948 | 41.871 | IM | 2007 | 222 | 22.7 | -19.55 |
| 112.130 | 43.650 | IM | 2003 | 62 | 22.7 | -18.63 |
| 111.920 | 43.530 | IM | 2003 | 62 | 22.8 | -19.64 |
| 111.262 | 41.225 | IM | 2007 | 462 | 22.8 | -20.60 |
| 109.307 | 40.634 | IM | 2007 | 167 | 22.8 | -19.75 |
| 108.569 | 40.901 | IM | 2007 | 167 | 22.8 | -19.12 |
| 111.634 | 41.181 | IM | 2007 | 462 | 22.8 | -17.81 |
| 109.344 | 40.572 | IM | 2007 | 167 | 22.9 | -15.58 |
| 113.339 | 42.393 | IM | 2007 | 90 | 22.9 | -18.08 |
| 113.339 | 42.393 | IM | 2007 | 90 | 22.9 | -20.10 |
| 112.049 | 42.016 | IM | 2007 | 222 | 22.9 | -18.79 |
| 113.570 | 43.830 | IM | 2005 | 87 | 22.9 | -17.56 |
| 113.050 | 43.750 | IM | 2004 | 74 | 23.0 | -20.11 |
| 106.455 | 40.742 | IM | 2007 | 167 | 23.0 | -20.50 |
| 106.562 | 40.813 | IM | 2007 | 167 | 23.0 | -13.50 |
| 116.720 | 45.330 | IM | 2001 | 54 | 23.1 | -23.70 |
| 115.900 | 44.400 | IM | 2001 | 54 | 23.1 | -22.23 |
| 115.430 | 44.000 | IM | 2001 | 54 | 23.1 | -23.68 |
| 112.202 | 42.171 | IM | 2007 | 222 | 23.2 | -20.77 |
| 115.520 | 43.920 | IM | 2001 | 54 | 23.2 | -22.61 |
| 106.725 | 40.801 | IM | 2007 | 167 | 23.2 | -19.17 |
| 116.930 | 45.770 | IM | 2001 | 54 | 23.3 | -20.16 |
| 117.670 | 44.550 | IM | 2000 | 57 | 23.4 | -22.19 |
| 115.900 | 44.130 | IM | 2001 | 54 | 23.5 | -22.17 |
| 112.358 | 42.330 | IM | 2007 | 222 | 23.5 | -18.93 |
| 113.050 | 43.750 | IM | 1999 | 57 | 23.6 | -19.49 |
| 112.932 | 42.446 | IM | 2007 | 90 | 23.7 | -18.57 |
| 112.808 | 42.535 | IM | 2007 | 90 | 23.9 | -20.42 |
| 113.050 | 43.750 | IM | 2001 | 54 | 23.9 | -20.87 |
| 112.700 | 42.780 | IM | 1999 | 57 | 24.0 | -20.86 |
| 116.930 | 44.430 | IM | 2000 | 57 | 24.0 | -22.34 |
| 112.780 | 43.730 | IM | 2005 | 87 | 24.2 | -18.17 |
| 115.830 | 44.920 | IM | 2000 | 57 | 24.2 | -21.44 |
| 111.970 | 43.380 | IM | 2004 | 74 | 24.2 | -18.12 |
| 115.800 | 44.620 | IM | 2000 | 57 | 24.3 | -21.95 |
| 111.630 | 43.370 | IM | 2004 | 74 | 24.3 | -22.07 |
| 115.430 | 44.000 | IM | 2000 | 57 | 24.4 | -20.12 |
| 116.680 | 43.570 | IM | 2000 | 57 | 24.4 | -20.25 |
| 111.700 | 43.470 | IM | 2004 | 74 | 24.4 | -21.40 |
| 111.900 | 43.500 | IM | 2004 | 74 | 24.4 | -19.21 |
| 111.920 | 43.530 | IM | 2004 | 74 | 24.5 | -17.64 |
| 116.500 | 44.250 | IM | 2000 | 57 | 24.5 | -17.51 |
| 115.900 | 44.400 | IM | 2000 | 57 | 24.5 | -22.24 |
| 116.600 | 43.780 | IM | 2000 | 57 | 24.6 | -22.91 |
| 112.470 | 43.680 | IM | 2001 | 54 | 24.6 | -20.14 |
| 108.569 | 40.901 | IM | 2000 | 110 | 25.2 | -19.70 |
| 111.630 | 43.370 | IM | 1999 | 57 | 25.2 | -21.28 |
| 112.470 | 43.680 | IM | 2000 | 57 | 26.2 | -19.90 |
| 107.496 | 47.353 | MN | 2006 | 51 | 17.7 | -22.41 |
| 107.317 | 47.573 | MN | 2006 | 51 | 18.1 | -22.68 |
| 107.317 | 47.573 | MN | 2006 | 51 | 18.1 | -22.98 |
| 107.645 | 47.048 | MN | 2006 | 51 | 18.2 | -22.16 |
| 107.691 | 47.748 | MN | 2006 | 145 | 18.3 | -23.59 |
| 108.020 | 47.721 | MN | 2003 | 116 | 18.3 | -21.58 |
| 107.680 | 47.025 | MN | 2006 | 51 | 18.3 | -21.78 |
| 107.702 | 47.033 | MN | 2006 | 51 | 18.3 | -21.55 |
| 108.020 | 47.721 | MN | 2006 | 145 | 18.3 | -22.11 |
| 107.227 | 47.755 | MN | 2006 | 145 | 18.3 | -21.53 |
| 107.717 | 47.010 | MN | 2006 | 51 | 18.4 | -21.35 |
| 108.491 | 47.701 | MN | 2006 | 145 | 18.5 | -22.81 |
| 109.500 | 47.500 | MN | 1996 | 31 | 18.6 | -22.92 |
| 109.500 | 47.500 | MN | 2003 | 41 | 18.9 | -22.98 |
| 109.500 | 47.500 | MN | 2003 | 41 | 18.9 | -22.82 |
| 109.130 | 47.656 | MN | 2006 | 145 | 18.9 | -22.28 |
| 109.130 | 47.656 | MN | 2006 | 145 | 18.9 | -20.91 |
| 113.871 | 45.308 | MN | 1996 | 31 | 19.0 | -22.38 |
| 109.619 | 47.455 | MN | 2003 | 41 | 19.0 | -20.99 |
| 113.379 | 45.417 | MN | 1996 | 31 | 19.1 | -17.72 |
| 113.354 | 45.423 | MN | 1998 | 37 | 19.1 | -20.40 |
| 108.035 | 46.715 | MN | 2006 | 38 | 19.2 | -20.78 |
| 108.083 | 46.707 | MN | 2006 | 38 | 19.3 | -20.35 |
| 109.500 | 47.500 | MN | 2006 | 38 | 19.3 | -21.57 |
| 113.386 | 46.879 | MN | 1996 | 31 | 19.3 | -22.35 |
| 107.227 | 47.755 | MN | 2004 | 114 | 19.4 | -22.70 |
| 109.619 | 47.455 | MN | 2006 | 38 | 19.4 | -21.85 |
| 115.325 | 45.906 | MN | 2003 | 41 | 19.5 | -21.90 |
| 115.325 | 45.906 | MN | 2006 | 38 | 19.5 | -21.66 |
| 113.333 | 47.031 | MN | 1998 | 37 | 19.5 | -17.56 |
| 115.204 | 45.847 | MN | 2006 | 38 | 19.6 | -19.84 |
| 109.996 | 47.382 | MN | 2006 | 38 | 19.6 | -19.75 |
| 110.513 | 47.385 | MN | 2006 | 38 | 19.7 | -21.46 |
| 114.664 | 46.021 | MN | 2006 | 38 | 19.7 | -20.37 |
| 114.664 | 46.021 | MN | 2003 | 41 | 19.8 | -20.80 |
| 114.863 | 45.670 | MN | 2006 | 38 | 19.9 | -21.35 |
| 114.858 | 45.640 | MN | 2006 | 38 | 19.9 | -19.56 |
| 114.858 | 45.640 | MN | 2006 | 38 | 19.9 | -19.41 |
| 111.274 | 47.248 | MN | 2003 | 41 | 20.0 | -21.04 |
| 114.134 | 46.102 | MN | 2006 | 38 | 20.0 | -20.60 |
| 114.109 | 46.125 | MN | 2006 | 38 | 20.0 | -20.07 |
| 111.274 | 47.248 | MN | 2006 | 38 | 20.1 | -21.49 |
| 113.386 | 46.879 | MN | 2006 | 38 | 20.2 | -21.32 |
| 109.130 | 47.656 | MN | 2005 | 122 | 20.2 | -23.23 |
| 113.620 | 46.336 | MN | 2006 | 38 | 20.2 | -20.16 |
| 109.619 | 47.455 | MN | 2005 | 37 | 20.2 | -21.69 |
| 109.500 | 47.500 | MN | 2004 | 39 | 20.3 | -21.90 |
| 110.513 | 47.385 | MN | 2005 | 37 | 20.3 | -22.09 |
| 113.871 | 45.308 | MN | 2003 | 41 | 20.3 | -21.55 |
| 113.420 | 46.165 | MN | 2006 | 38 | 20.4 | -20.48 |
| 108.464 | 46.323 | MN | 2006 | 38 | 20.4 | -21.29 |
| 111.798 | 47.040 | MN | 2006 | 38 | 20.4 | -20.44 |
| 107.691 | 47.748 | MN | 2005 | 122 | 20.4 | -23.04 |
| 114.858 | 45.640 | MN | 2004 | 39 | 20.5 | -20.33 |
| 115.204 | 45.847 | MN | 2005 | 37 | 20.5 | -21.55 |
| 113.620 | 46.336 | MN | 2003 | 41 | 20.5 | -20.46 |
| 113.620 | 46.336 | MN | 2003 | 41 | 20.5 | -19.76 |
| 112.338 | 46.820 | MN | 2006 | 38 | 20.5 | -21.22 |
| 112.327 | 46.824 | MN | 2006 | 38 | 20.5 | -20.57 |
| 113.871 | 45.308 | MN | 2006 | 38 | 20.6 | -21.05 |
| 113.871 | 45.308 | MN | 2006 | 38 | 20.6 | -22.91 |
| 112.417 | 46.500 | MN | 2003 | 41 | 20.8 | -20.60 |
| 111.274 | 47.248 | MN | 2005 | 37 | 20.9 | -22.19 |
| 114.863 | 45.670 | MN | 2005 | 37 | 20.9 | -20.87 |
| 113.379 | 45.417 | MN | 2006 | 38 | 20.9 | -17.63 |
| 113.354 | 45.423 | MN | 2006 | 38 | 20.9 | -18.51 |
| 113.207 | 45.409 | MN | 2006 | 38 | 21.0 | -19.78 |
| 108.491 | 47.701 | MN | 2002 | 114 | 21.2 | -21.79 |
| 108.872 | 46.009 | MN | 2006 | 38 | 21.3 | -20.53 |
| 113.620 | 46.336 | MN | 2002 | 39 | 21.4 | -22.04 |
| 111.798 | 47.040 | MN | 2005 | 37 | 21.4 | -20.16 |
| 109.619 | 47.455 | MN | 2000 | 43 | 21.4 | -20.64 |
| 114.863 | 45.670 | MN | 2001 | 37 | 21.5 | -21.91 |
| 109.996 | 47.382 | MN | 2002 | 39 | 21.5 | -22.83 |
| 112.639 | 45.206 | MN | 2006 | 38 | 21.7 | -18.45 |
| 113.620 | 46.336 | MN | 2005 | 37 | 21.7 | -19.77 |
| 112.141 | 45.191 | MN | 2003 | 41 | 21.8 | -20.75 |
| 113.420 | 46.165 | MN | 2005 | 37 | 21.8 | -20.61 |
| 111.827 | 47.052 | MN | 2004 | 39 | 21.8 | -19.46 |
| 113.620 | 46.336 | MN | 2004 | 39 | 21.9 | -19.04 |
| 110.801 | 44.730 | MN | 1996 | 18 | 21.9 | -21.50 |
| 113.420 | 46.165 | MN | 2004 | 39 | 22.0 | -21.44 |
| 109.422 | 45.806 | MN | 2006 | 38 | 22.0 | -20.40 |
| 109.422 | 45.806 | MN | 2006 | 38 | 22.0 | -17.37 |
| 113.871 | 45.308 | MN | 1999 | 42 | 22.1 | -22.33 |
| 113.386 | 46.879 | MN | 2004 | 39 | 22.1 | -20.65 |
| 111.278 | 44.028 | MN | 1996 | 18 | 22.1 | -19.71 |
| 112.945 | 46.764 | MN | 2004 | 39 | 22.1 | -19.87 |
| 112.327 | 46.824 | MN | 2004 | 39 | 22.1 | -19.28 |
| 112.945 | 46.764 | MN | 2001 | 37 | 22.1 | -20.64 |
| 113.379 | 45.417 | MN | 1999 | 42 | 22.1 | -21.46 |
| 112.141 | 45.191 | MN | 2006 | 38 | 22.1 | -18.22 |
| 112.338 | 46.820 | MN | 2004 | 39 | 22.1 | -21.46 |
| 111.849 | 43.723 | MN | 1996 | 18 | 22.2 | -17.78 |
| 108.872 | 46.009 | MN | 2004 | 39 | 22.2 | -21.69 |
| 111.798 | 47.040 | MN | 2000 | 43 | 22.4 | -21.71 |
| 112.338 | 46.820 | MN | 2002 | 39 | 22.4 | -21.26 |
| 112.417 | 46.500 | MN | 2002 | 39 | 22.5 | -17.96 |
| 108.872 | 46.009 | MN | 2001 | 37 | 22.6 | -21.54 |
| 113.333 | 47.031 | MN | 2000 | 43 | 22.7 | -20.18 |
| 97.333 | 47.833 | MN | 1999 | 129 | 22.7 | -21.96 |
| 113.333 | 47.031 | MN | 1999 | 42 | 22.8 | -20.90 |
| 108.872 | 46.009 | MN | 2002 | 39 | 22.9 | -20.87 |
| 109.641 | 45.264 | MN | 2006 | 38 | 23.0 | -17.73 |
| 112.141 | 45.191 | MN | 2002 | 39 | 23.2 | -20.87 |
| 113.620 | 46.336 | MN | 2000 | 43 | 23.2 | -19.71 |
| 110.801 | 44.730 | MN | 2003 | 15 | 23.4 | -17.97 |
| 110.801 | 44.730 | MN | 2003 | 15 | 23.4 | -15.20 |
| 113.420 | 46.165 | MN | 2000 | 43 | 23.4 | -22.19 |
| 110.589 | 44.812 | MN | 2003 | 15 | 23.4 | -17.62 |
| 108.872 | 46.009 | MN | 2000 | 43 | 23.7 | -21.60 |
| 108.872 | 46.009 | MN | 2000 | 43 | 23.7 | -21.22 |
| 110.997 | 44.591 | MN | 2006 | 11 | 23.7 | -19.01 |
| 111.141 | 44.449 | MN | 2006 | 11 | 23.7 | -18.70 |
| 110.801 | 44.730 | MN | 2006 | 11 | 23.7 | -17.74 |
| 110.801 | 44.730 | MN | 2006 | 11 | 23.7 | -18.65 |
| 111.126 | 44.368 | MN | 2006 | 11 | 23.8 | -18.83 |
| 111.126 | 44.368 | MN | 2006 | 11 | 23.8 | -18.62 |
| 110.589 | 44.812 | MN | 2006 | 11 | 23.8 | -17.65 |
| 111.476 | 43.988 | MN | 2006 | 11 | 23.8 | -18.63 |
| 111.374 | 44.048 | MN | 2006 | 11 | 23.9 | -19.58 |
| 111.702 | 43.807 | MN | 2006 | 11 | 23.9 | -19.64 |
| 111.793 | 43.760 | MN | 2006 | 11 | 23.9 | -17.68 |
| 111.747 | 43.779 | MN | 2006 | 11 | 23.9 | -18.42 |
| 111.479 | 43.937 | MN | 2006 | 11 | 23.9 | -19.28 |
| 111.700 | 43.798 | MN | 2006 | 11 | 23.9 | -19.63 |
| 111.849 | 43.723 | MN | 2006 | 11 | 23.9 | -19.49 |
| 111.830 | 43.726 | MN | 2006 | 11 | 23.9 | -19.21 |
| 111.409 | 43.951 | MN | 2006 | 11 | 23.9 | -18.91 |
| 111.278 | 44.028 | MN | 2006 | 11 | 23.9 | -18.90 |
| 111.128 | 44.100 | MN | 2006 | 11 | 23.9 | -18.64 |
| 110.236 | 44.861 | MN | 2006 | 38 | 24.0 | -16.35 |
| 110.236 | 44.861 | MN | 2006 | 38 | 24.0 | -16.24 |
| 111.514 | 43.684 | MN | 2006 | 11 | 24.0 | -18.43 |
| 113.354 | 45.423 | MN | 2000 | 43 | 24.2 | -21.00 |
| 111.126 | 44.368 | MN | 2002 | 13 | 24.5 | -19.51 |
| 111.126 | 44.368 | MN | 2002 | 13 | 24.5 | -17.78 |
| 110.801 | 44.730 | MN | 2002 | 13 | 24.5 | -17.65 |
| 110.589 | 44.812 | MN | 2002 | 13 | 24.6 | -19.33 |
| 110.589 | 44.812 | MN | 2002 | 13 | 24.6 | -17.17 |
| 111.141 | 44.449 | MN | 2004 | 16 | 24.7 | -20.75 |
| 110.801 | 44.730 | MN | 2001 | 14 | 24.7 | -19.37 |
| 111.700 | 43.798 | MN | 2002 | 13 | 24.8 | -20.42 |
| 111.747 | 43.779 | MN | 2002 | 13 | 24.8 | -20.51 |
| 110.801 | 44.730 | MN | 2005 | 15 | 25.0 | -17.41 |
| 110.589 | 44.812 | MN | 2005 | 15 | 25.1 | -19.73 |
| 111.278 | 44.028 | MN | 2001 | 14 | 25.2 | -20.49 |
| 111.374 | 44.048 | MN | 2005 | 15 | 25.2 | -21.43 |
| 111.747 | 43.779 | MN | 2005 | 15 | 25.2 | -17.66 |
| 111.278 | 44.028 | MN | 2005 | 15 | 25.3 | -21.64 |
| 111.479 | 43.937 | MN | 2000 | 20 | 27.0 | -18.84 |
| 111.278 | 44.028 | MN | 2000 | 20 | 27.0 | -19.21 |
